# Quantifying social contacts in a household setting of rural Kenya using wearable proximity sensors

**DOI:** 10.1140/epjds/s13688-016-0084-2

**Published:** 2016-06-14

**Authors:** Moses C Kiti, Michele Tizzoni, Timothy M Kinyanjui, Dorothy C Koech, Patrick K Munywoki, Milosch Meriac, Luca Cappa, André Panisson, Alain Barrat, Ciro Cattuto, D James Nokes

**Affiliations:** KEMRI - Wellcome Trust Research Programme, Kilifi, Kenya; Data Science Laboratory, ISI Foundation, Via Alassio 11/c, Torino, 10126 Italy; School of Mathematics, The University of Manchester, Manchester, UK; Bitmanufaktory Ltd, Cambridge, UK; Aix-Marseille Université, Université de Toulon, CNRS, CPT, UMR 7332, Marseille, 13288 France; School of Life Sciences and WIDER, University of Warwick, Coventry, UK

**Keywords:** households, contact patterns, contact networks, wearable proximity sensors, respiratory infections, infectious disease control

## Abstract

Close proximity interactions between individuals influence how infections spread. Quantifying close contacts in developing world settings, where such data is sparse yet disease burden is high, can provide insights into the design of intervention strategies such as vaccination. Recent technological advances have enabled collection of time-resolved face-to-face human contact data using radio frequency proximity sensors. The acceptability and practicalities of using proximity devices within the developing country setting have not been investigated.

We present and analyse data arising from a prospective study of 5 households in rural Kenya, followed through 3 consecutive days. Pre-study focus group discussions with key community groups were held. All residents of selected households carried wearable proximity sensors to collect data on their close (<1.5 metres) interactions. Data collection for residents of three of the 5 households was contemporaneous. Contact matrices and temporal networks for 75 individuals are defined and mixing patterns by age and time of day in household contacts determined. Our study demonstrates the stability of numbers and durations of contacts across days. The contact durations followed a broad distribution consistent with data from other settings. Contacts within households occur mainly among children and between children and adults, and are characterised by daily regular peaks in the morning, midday and evening. Inter-household contacts are between adults and more sporadic when measured over several days. Community feedback indicated privacy as a major concern especially regarding perceptions of non-participants, and that community acceptability required thorough explanation of study tools and procedures.

Our results show for a low resource setting how wearable proximity sensors can be used to objectively collect high-resolution temporal data without direct supervision. The methodology appears acceptable in this population following adequate community engagement on study procedures. A target for future investigation is to determine the difference in contact networks within versus between households. We suggest that the results from this study may be used in the design of future studies using similar electronic devices targeting communities, including households and schools, in the developing world context.

**Electronic Supplementary Material:**

The online version of this article (doi:10.1140/epjds/s13688-016-0084-2) contains supplementary material.

## Introduction

Close social contacts drive the spread of respiratory infections that are transmitted by respiratory droplets or saliva [1]. Improved characterization of these social contacts should lead to an improved understanding of the dynamics of infectious diseases with this mode of transmission within human communities, and increasingly, such data is utilized within predictive transmission dynamic models [2–14]. The collection of close contact data has however many challenges [15–18]. The most important consists in defining the form of contact required to effect transmission [19], and, in turn, the methodology that can be employed to collect unbiased data on such behaviour.

In this context, the standard definition for a close contact is co-location with an individual such that both have a conversation without raising voices, or having a direct (physical) contact that entails skin-to-skin touch between the individuals [3]. The recording of such behaviour has primarily been performed using daily contact diaries (self or 3rd party completed [9, 14, 16, 20, 21]), or retrospective questionnaires on contacts made [2, 12, 17, 22, 23]. The collection of contact data using these methods has moreover primarily focused on populations in developed countries [2, 3, 9, 10, 16, 18, 24], rather than in developing [12, 14, 21] countries. The level of respiratory infectious disease burden in low-income countries suggests that increased attention on developing country communities is justified in the future.

The contact diary has been the mainstay of studies recording contact data, in which, for example, respondents record whom they contact (age, gender) and how often, whether there was skin-to-skin contact, the location (home, work, school, other), and how long the encounter lasted. Several limitations on accurate collection of representative data have been identified such as recall bias [16], low compliance and illiteracy [21], and differences in definitions [3, 14, 25]. Completion of diaries is a time consuming occupation, may alter behaviour, and require prior user training. Alternatives to collecting contact data via diary questionnaires are Web-based interfaces [2, 15] or focus group discussions [25], with the methods adapted to suit the context. Alternatively, synthetic contact matrices can be generated from co-location data using time-use [26] or demographic data [8, 27]. All these methods are of limited value in defining networks of contacts.

More recently, a host of proximity-sensing technologies have paved the way to automated collection of social proximity data: Bluetooth-enabled smartphones [28, 29], radio beacons [30], wearable radio frequency (RF) devices [31], and more. In particular, proximity-sensing wearable devices (henceforth referred to as ‘tags’) achieve low cost, have simple operational constraints, and can be tuned to detect proximity interactions at less than 1 meter separation distance every few seconds [32]. These proximity events are deemed relevant to direct (through physical contact) or indirect (via aerosols) spread of infections. When worn by participants, they provide data on temporal dynamic interaction patterns in real-world environments, for example, schools [33, 34], hospitals [35, 36] and conferences [37]. Data from these studies highlight important network properties, such as the presence of superspreaders (nodes) who are more likely to spread infections compared to others based on the number and duration of their interactions. Schools highlight age and school class assortativity as important to infection control [34, 38, 39], while hospital research identified nurses as having the most potential to transmit infections to patients [40].

Investigation of contact patterns in high-contact settings such as schools and households is of paramount importance in epidemiology [41]. In particular, households are considered hubs of infection spread [42] because of high frequency and long duration contacts with high proportion of physical interactions, combined with a high degree of clustering between members of the same household [43]. Furthermore, introduction of infection into households and onward dissemination from households is dependent on the connectivity of households with other groups (such as schools and workplaces, as well as other households) within a community. However, there is a distinct lack of information, in both developed and developing countries, on contact patterns at the household level, and on the role of the household network structure in shaping disease transmission. Furthermore, there is little information from the low resource setting on the acceptability of use, methodology of implementation, and performance, of electronic tracking methods, both proximity sensors and GPS locators.

The present study offers a simultaneous assessment of intra- and, to a lesser extent, inter-household social contact patterns. We report the number and duration of contacts and the influence of age and day, as well as the temporal structure of the networks. We also report on the experience of undertaking the study in a low resource setting, from the researcher, the community and the individual participant perspectives, including the challenges and limitations. Although the data set is limited in size, since this was principally a feasibility study, it represents a first step in developing the use of electronic proximity and tracking in a rural developing country setting which we hope will provide the basis for more detailed and expansive studies.

## Data and methods

### Study design, context and data collection

**Figure 1:**
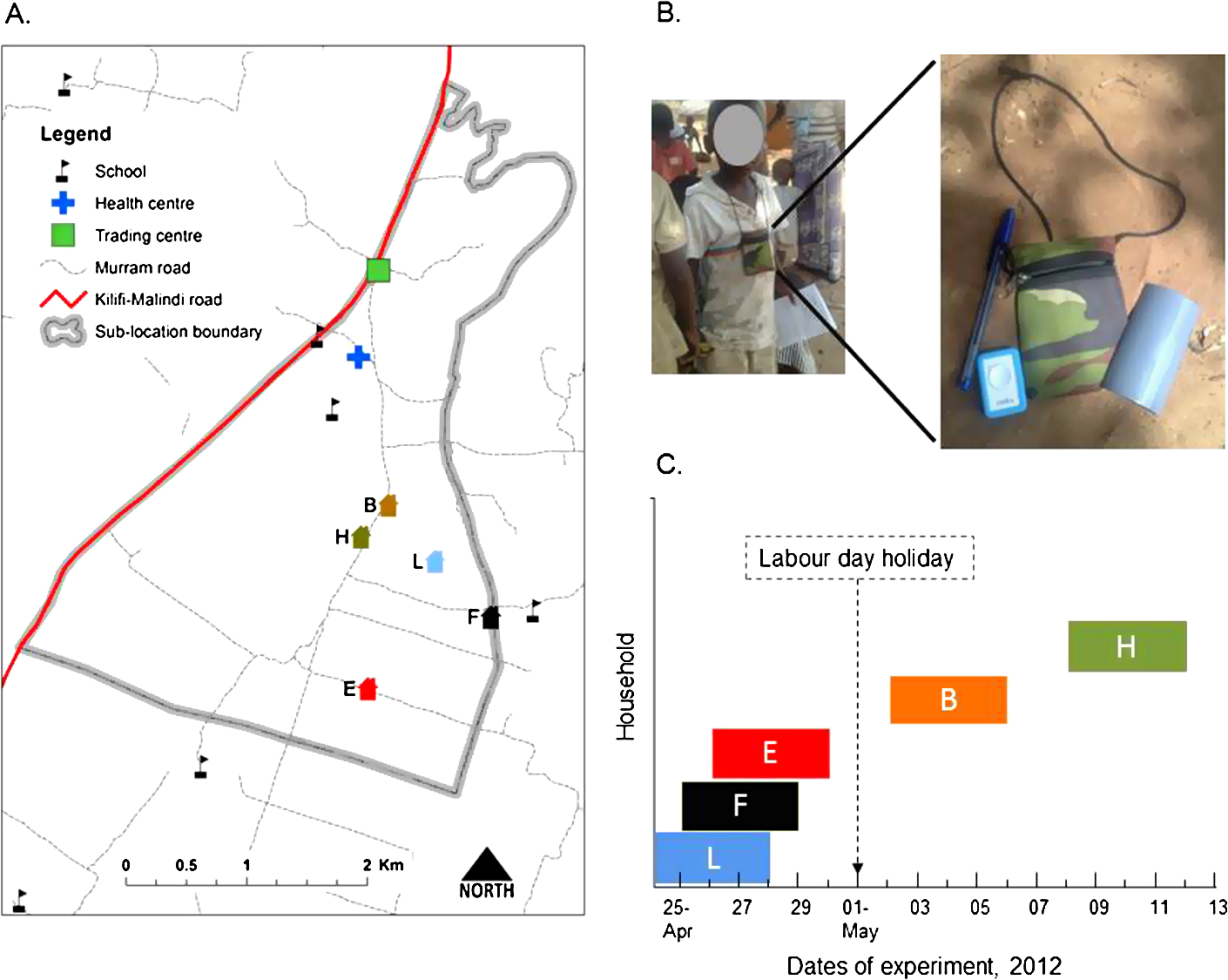
**Study design.** Panel **A** shows the selection of households within the study area. Panel **B** shows a child wearing the tag worn with a lanyard around the neck. Panel **C** shows data collection over time across the households, highlighting E-F-L in which data was collected concurrently.

The study was conducted in the Matsangoni sub-location within the Kilifi Health and Demographic Surveillance Site (KHDSS), coastal Kenya [44]. Five households (Figure 1, panel A) were selected at random from a group of 50 households that had earlier participated in a study to investigate ‘who acquires infection from whom’ (WAIFW) [45]. A household was defined as all people who eat from the same kitchen. In this rural setting, a household encompasses several related families living in distinct houses within the same compound and reporting to one head. Participants were grouped into 6 age groups assumed to approximate key social or behavioral groups: <1 (infant), 1-5 (pre-school), 6-15 (primary school), 16-19 (secondary school), 20-49 (adults), and ≥50 (elderly) years.

Prior to the study, 5 focus group discussions were conducted in the study area focusing on four thematic areas: acceptability of the tags for data collection, participants and non-participants perceptions of tags, privacy concerns and length of time to carry tags. The groups were composed of primary school students (class 4-8, approximate age range 10-17 y), secondary school students (form 1-4, age range 15-21 y) and kindergarten teachers (age range 23-55 y). The last group was separate male and female Kenya Medical Research Institute (KEMRI) Community Representatives (KCR, age range 20-50 y). KCRs are a network of community-elected individuals who provide feedback on research activities to and from KEMRI and the community [46] and are recognized as a key informant group for research activities. Community sensitization commenced with seeking permission from the local administrative officers in Matsangoni. At each household, the head gave the initial approval for the research team to engage the rest of the members. For practical reasons, the study procedures were explained to all available members simultaneously in a manner reflecting the developmental age of the individuals, and follow-ups were made appropriately for those who missed the joint sessions. Active engagement for children below 14 years old was enhanced by use of information, education and content materials in form of colouring books containing health messages. Teenage siblings and adults received more detailed study fliers and were given a toll-free study number to call in case of further questions.

Data were collected using the platform developed by the SocioPatterns collaboration project (a European consortium of institutions and investigators focused on social dynamics, http://www.sociopatterns.org). The sensors are wearable devices that exchange ultra-low power radio packets and can detect close proximity of individuals wearing them [32]. The infrastructure of the SocioPatterns sensing platform has been further described in several papers [34–36]. For this study, the tags were tuned to exchange data packets only when located within <1.5 metres, suggesting a dyadic conversation or skin-to-skin touch such as a handshake. A close proximity ‘contact’ between two individuals with tags occurred when at least one data packet was exchanged in a 20 second window. Once a contact was established, it was considered ongoing until no packets were exchanged for 20 consecutive seconds. Several 20-second contact windows are aggregated to give the duration of one contact between different individuals. Participants were asked to wear the tags with a lanyard on the chest. Since the radiofrequency used to sense proximity cannot propagate through a human body, this enabled detection of face-to-face proximity relations. All data was collected and stored in the internal memory of the wearable devices and downloaded to a computer for post-processing and analysis.

Participants were approached for consent at the households by trained fieldworkers. Before a participant was given a tag (Figure 1, panel B), it was reset to clear its memory. Note that participants were not trained on how to perform this procedure. The tags were enclosed in a pouch with lanyard and given to each participant to wear around the neck. Participants wore a tag for five days; however, each household received tags on separate days, *e.g.*, household L started on April 24th at 2 pm to 28th, household F from 25th to 29th and household E from 26th to 30th. Residents of these three households (E, F, L) carried the tags during an overlapping time window (Figure 1, panel C). On the fifth day of data collection, fieldworkers went to each household to collect the tags.

### Data analysis

The data collected by the tags provide a high-resolution measurement of the contact patterns between household members at the temporal scale of 20 seconds. First, we extracted and cleaned the data separately for each participant, identifying corrupted sensors (no data available) or anomalous signals (such as continuous bursts of data) in the contact measurements. In order to make the contact dataset comparable across households, we discarded data collected on the first and fifth day and considered only contacts collected over 3 consecutive days, from 6 am to 8 pm, for each household. Night contacts, collected from 8 pm to 6 am, were disregarded from the analysis because most tags were not worn by the participants during night time. Only individuals that had a complete contact record for 3 consecutive days were considered in the analysis. Measurements obtained from sensors that were restarted or interrupted before the end of the experiment were not included. Table 1 reports a full description of age and gender of all participants by household, indicating the individuals whose contact records were excluded from the data analysis.

**Table 1:** **Age and gender distribution of participants by household**

	**Age 0-5 (M, F)**	**Age 6-14 (M, F)**	**Age 15-19 (M, F)**	**Age 20-49 (M, F)**	**Age** **≥50** **(M, F)**	**Total (M, F)**
*Included in data analysis*						
Household B	4 (1, 3)	4 (1, 3)	1 (0, 1)	4 (1, 3)	2 (0, 2)	15 (3, 12)
Household E	3 (3, 0)	7 (3, 4)	0	7 (3, 4)	0	17 (9, 8)
Household F	1 (0, 1)	1 (1, 0)	2 (2, 0)	3 (1, 2)	1 (0, 1)	8 (4, 4)
Household H	6 (1, 5)	13 (4, 9)	2 (1, 1)	6 (1, 5)	2 (0, 2)	29 (7, 22)
Household L	1 (1, 0)	1 (0, 1)	1 (1, 0)	3 (0, 3)	0	6 (2, 4)
*Excluded from data analysis* ^$^						
Household B	4 (1, 3)	1 (0, 1)	1 (1, 0)	4 (2, 2)	0 (0, 0)	12 (5, 7)^∗^
Household E	0	0	0	1 (1, 0)	1 (0, 1)	2 (1, 1)
Household F	0	0	0	0	1 (1, 0)	1 (1, 0)
Household H	3 (1, 2)	3 (1, 2)	0	4 (2, 2)	0	10 (4, 6)
Household L	0	0	0	0	0	0

^$^Reasons for exclusion of data are described in the Data and methods section.

^∗^The age of two members of household B excluded from the analysis was not recorded.

We first compute the number of contact events recorded by each individual and the statistical distribution of the duration of contact events, and then aggregate such statistics by age and household. Contact events can be of two types:

I. contacts between members of the same household (available for all the households);
II. contacts between members of different households (available only for households E, F, and L).

Given the different nature and frequency of the two types of events, we disaggregated the dataset by type of contacts and analyzed the two sets separately.

Furthermore, we generate the aggregated contact networks on a daily scale and on the full experimental time period (3 days). Nodes of the networks are individuals, while an edge indicates the presence of at least one recorded contact event between the two involved individuals during the aggregation time window. Given a contact network, we define the following quantities:

- the degree $k_{i}$ of a node *i* represents the number of distinct individuals with whom individual *i* has been in contact during the time window;
- the weight $n_{ij}$ of an edge between nodes *i* and *j* is the number of contact events recorded between these individuals during the time window;
- the weight $w_{ij}$ of an edge between nodes *i* and *j* is the cumulative duration of the $n_{ij}$ contacts recorded during the time window between the two individuals.

Network edges are undirected and weights on the edges are symmetric ($n_{ij} = n_{ji}$, $w_{ij} = w_{ji}$). We study the statistical distributions of the degrees and weights of the contact networks and extract from them age stratified contact matrices. We characterize the statistical distributions by computing their average and the squared coefficient of variation ($\mathit{CV}^{2}$), defined as the ratio between the standard deviation and the mean of the distribution. The squared coefficient of variation is used to distinguish between high-variance distributions ($\mathit{CV}^{2} >1$) and low-variance distributions ($\mathit{CV}^{2} <1$).

We compare age-stratified contact matrices extracted from the empirical contact networks with synthetic matrices generated by an algorithmic approach [8]. To quantify differences between matrices, we compute the basic reproductive number $R_{0}$ defined as the dominant eigenvalue of the next generation matrix [5], which is equivalent to the dominant eigenvalue of the contact matrix up to a constant. Under the null hypothesis of equal contact matrices, the ratio of estimates of $R_{0}$ is expected to equal 1. For each matrix comparison, we assess the statistical significance of any deviation from the null hypothesis by calculating 95% confidence intervals based on a nonparametric bootstrap with 1,000 samples of the empirical contact data. In practice, we sample 1,000 times the empirical data and compare each sampled empirical matrix of one household to the corresponding synthetic matrix, whose generation process cannot be randomized, obtaining one value of the $R_{0}$ ratio from each comparison.

We analyse the temporal variability of the contact networks aggregated on a daily scale by measuring the node loyalty [47] and the similarity between the neighborhoods of a node in two different days [23]. The loyalty *θ* measures the fraction of preserved neighbors of a node for a pair of two network configurations at time $t_{1}$ and $t_{2}$. If the set of neighbors of node *i* at time *t* is denoted as $\varGamma_{i}^{t}$, then the loyalty $\theta_{i}^{t_{1}, t_{2}}$ is given by the Jaccard index [34]:

$$\theta_{i}^{t_{1}, t_{2}} =\biggl\vert \frac{\varGamma_{i}^{t_{1}} \cap \varGamma_{i}^{t_{2}} }{\varGamma_{i}^{t_{1}} \cup \varGamma_{i}^{t_{2}}}\biggr\vert . $$

The loyalty takes values between 0 and 1, with $\theta =0$ indicating that no neighbors are retained from time $t_{1}$ to time $t_{2}$, and $\theta =1$ indicating that the set of neighbors is exactly the same in the two configurations. The definition of loyalty does not take into account the presence of weights on the edges. To measure the similarity between neighborhoods of nodes, also taking into account the time spent in contact, we consider the cosine similarity [34], defined as:

$$\operatorname{sim}( i )= \frac{\sum_{j} ( w_{ij,1} w_{ij,2} )}{\sqrt{( \sum_{j} w_{ij,1}^{2} )} \sqrt{( \sum_{j} w_{ij,2}^{2} )}}, $$

where $w_{ij,1}$ and $w_{ij,2}$ are the weights on the edge $i \leftrightarrow j$ measured at time $t_{1}$ and $t_{2}$, respectively. The cosine similarity takes values between 0 and 1, where 1 corresponds to the case when *i* had contacts in the two time windows with exactly the same individuals and spending the same fraction of time in proximity with each of them. If the sets of neighbors are completely different in the two configurations, the cosine similarity is zero.

### Ethical review and consent

The Kenya Ethical Review Committee (KEMRI/RES/7/3/1) and the Biomedical and Social Ethics Review Committee of the University of Warwick (119-05-2011) approved the study. Written informed consent was sought from participants aged ≥18 years and from parents or guardians for those aged <18 years. Infants (<12 months of age) did not participate in this study.

## Results

### Focus group discussions

Focus group discussions were held with a total of 52 respondents (9 adult males, 6 adult females, 10 KEMRI Community Representatives, 15 primary school students and 12 secondary school students). Of the non-students, 3 had no education, 12 primary school education, 8 secondary school education and 1 college education. Their main economic activity was small-scale business, farming, and casual labour or employed as a teacher (1) or in the hotel industry (2). Participants agreed in as much as the community would welcome the research, however, it was made clear that a more thorough explanation of the study tools and procedures was necessary. For instance, clarification on whether the tags detected a respiratory infection in addition to proximity between tags. Most participants thought that the devices were relatively small making then comfortable to be carried for a number of days. Some mentioned that a shorter period would make it convenient for them to carry the devices, while others argued that a shorter duration might not give an accurate picture of their regular movement and contact patterns. Despite the size of the tag, there were concerns regarding dangers posed by the lanyard around the neck or children getting hurt when the tag was pressed against the chest. Different ways of carrying the devices were proposed, such as in the shirt or trouser pocket for men, attached to blouses for women, or encased in a pouch hung around the neck. This concealment would ensure that participants did not lose or tamper with the tags and would minimize questions from non-participants. From these discussions, it was agreed that the devices would be inserted in pouches and carried around the neck for a period of 4 days and involve all children especially due to the paucity of data in this age group and their importance in the spreading of infections at schools and households.

### Baseline characteristics

Out of 161 listed residents, 100 (65% female) were enrolled and assigned tags. One infant was not enrolled since the tag was potentially dangerous due to its size and shape. Other individuals, especially adults aged >15 years, were not available at the households during the entire study period mainly due to work and school. The median age was 16.9 years (IQR 6.9-29.7) and household size ranged from 6 to 40 participants. Participants were regrouped into 5 age groups, instead of 6, by merging all aged <6 year olds. Data were collected over four days for each household within the period 24th April 2011 to 11th May 2011. No data was collected on May 1st as it was a gazetted public holiday, as well as on three other separate days to enable recharging of the electronic devices and monitoring of study progress. During the data analysis, we observed a number of tags displaying anomalies such as spikes in their activity records. We also excluded all data collected on the first day from all tags. Overall, these tags correspond to 25% of the total and, eventually, only data from 75 tags were used in the analysis reported here (see Table 1).

We recorded 32,943 contact events in 15 days. A total of 32,425 (98%) contacts were recorded between members of the same household, and 218 contact events between members of different households. Table 2 displays the summary statistics of the contacts recorded between members of the same household. We recorded an average number of 144 contact events per person, per day, with a standard deviation of 141 contact events ($\mathit{CV}^{2} =0.96$), thus very close to the characteristics of an exponential distribution ($\mathit{CV}^{2} =1$). School children aged 6-14 years recorded the highest total number of contacts, with those aged 15-19 years recording the lowest. However, children aged 0-5 years have the highest average number of daily contacts across all ages. Disaggregating the contacts by gender, we found no statistically significant difference between the contact distributions of males and females, using a two-sample Kolmogorov-Smirnov test ($p\mbox{-value} =0.47$); males reported an average of 186 daily contacts per individual ($\mathit{CV}^{2} =0.99$), and females had an average of 123 daily contacts per individual ($\mathit{CV}^{2} =0.76$).

**Table 2:** **Statistics of contacts within households**

**Age class**	**Number of individuals**	**Total number of contacts**	**Number of daily contacts per individual (95% RR)**	**Average degree** ^∗^
0-5	15	10,804	240 (80-477)	17.5
6-14	26	15,462	198 (21-576)	18.3
15-19	6	607	34 (9-92)	11.7
20-49	23	4,835	70 (8-231)	12.2
≥50	5	717	48 (9-93)	12.6
**Gender**				
Male	25	13,933	186 (8-578)	15.0
Female	50	18,492	123 (8-365)	15.5
*Total*	75	32,425	144 (8-496)	15.3

^∗^Arithmetic mean degree (see Data and methods section).

**Figure 2:**
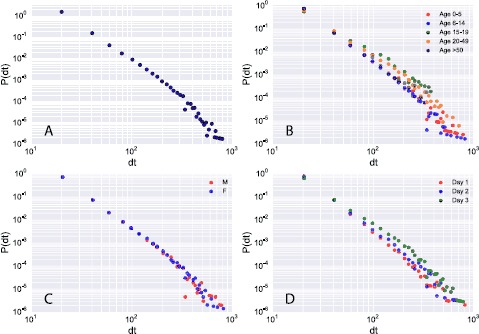
**Distribution of contact duration.** Each panel shows the probability density distribution ($\mathrm{P}(\mathrm{dt})$) of contact durations (dt) measured over the whole experimental period (3 consecutive days for each household). The full distribution is shown in panel **A**, while in the other panels it is stratified by age (**B**), gender (**C**) and day of study (**D**).

Figure 2 shows the probability distribution of person-to-person contact durations within households for all participants (panel A) and further stratified by age (B), gender (C) and day of study (D). The average contact duration measured on all contact events is 38 seconds with about 1% of contacts exceeding 5 minutes (the squared coefficient of variation of the full distribution is $\mathit{CV}^{2} =1.5$). There is a similar distribution across all age groups, but most of the longest contact durations are measured among children aged <14 years, who are also the most represented age group in the dataset. Contacts between individuals aged 15-19 and >50 years do not have very long durations.

### Contact matrices

**Figure 3:**
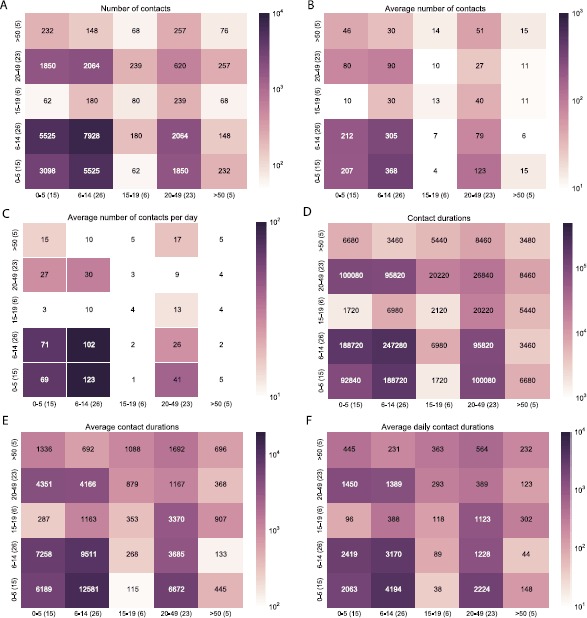
**Contact matrices giving the number (A)-(C) and cumulative duration (D)-(F) of contacts within households by age.** The first column shows the total number of contacts (**A**) and durations (**D**) that individuals of age *i* (column-index) had with individuals of age *j* (row-index) over 3 days. The second column shows the average (arithmetic mean) number of contacts (**B**) and duration (**E**) of an individual of age *i* with individuals of age *j*. The third column shows matrices B and E normalized by the duration of the study to obtain the average number of contacts per day (**C**) and duration (**F**) between an individual aged *i* and all individuals of age *j*. Labels on the *x* and *y* axes report the age groups and the number of individuals in each group, in parenthesis. Durations are reported in seconds.

We generated contact matrices based on number and duration of contacts by age (Figure 3) and stratified by household (see Figures S1-S5 in Additional file 1). Figure 3 shows the total number of contacts (panel A) and the cumulative durations (panel D) between age groups, computed over the study duration and considering only contacts within households. Panels B and E show the average number of contacts and cumulative durations per individual, taking into account the number of participants in each age group and thus yielding asymmetric matrices. Panels C and F show the daily average number of contacts and durations per participant (average entries divided by 3). Matrices of number and duration of contacts reveal different levels of assortativity for the different age groups. Age assortativity is observed in children aged <14 years, with children aged 0-5 years having more contacts with older participants aged 6-14 years. Disassortativity is instead observed for older age groups, with adults aged 20-49 years spending most of their time with children aged 0-5, which is easily explained by their parenthood, and teenagers aged 15-19 spending most of their time with adults. The least numbers of interactions are observed between pre-school children and teenagers and between school children (6-14) and the elderly (≥50).

To assess whether such contact matrices could be inferred by a simple random mixing assumption, we generated a contact matrix separately for each household by using the method proposed by Fumanelli *et al.* [8] (see Additional file 1 for details). We compute such synthetic contact matrix by assuming that the contact frequency between age groups is simply proportional to the number of individuals in the two groups in the household. In Figures S6-S10, in Additional file 1, we show a side-by-side comparison of the contact matrices measured by proximity sensors and the synthetic matrices based on the age structure of each household. For the sake of comparison, the entries of the sensor-based matrices are normalized by the total number of contacts recorded in the household, thus yielding the contact fraction measured between age groups. It is immediate to see that sensor based contact matrices display higher heterogeneities in the distributions of contacts between age groups, with differences with respect to the synthetic matrices that are more significant as the household gets smaller. For instance, contacts between children tend to be higher than expected by random mixing, as observed in household E (Figure S7), household F (Figure S8) and household H (Figure S9). Also, sensor based matrices show higher than expected contact frequencies between children and adults, such as in household L (Figure S10). On the other hand, mixing between adults tend to be overestimated by synthetic matrices, such as in households E and F.

While qualitative differences can be assessed by visual inspection, a more quantitative analysis is needed to identify epidemiologically relevant differences between matrices. To this aim, we compared contact matrices using the basic reproductive number $R_{0}$, following the approach of Hens *et al.* [5]. Comparing the contact patterns measured by sensors with the synthetic matrices, we found an $R_{0}$ ratio significantly different from 1 for all the households (see Table 3) and systematically larger than 1, indicating that a random mixing assumption may not be adequate to describe contact patterns within households.

**Table 3:** **Basic reproduction numbers for sensor-based and synthetic contact patterns**

**Household**	$\boldsymbol{R}_{\boldsymbol{0}}$ **ratio**	**95% confidence interval** ^∗^
B	1.14	1.13,1.16
E	1.37	1.36,1.38
F	1.12	1.10,1.15
H	1.22	1.21,1.23
L	1.09	1.07,1.12

^∗^Bootstrap-based 95% confidence interval (see Data and methods section).

### Contact network

**Figure 4:**
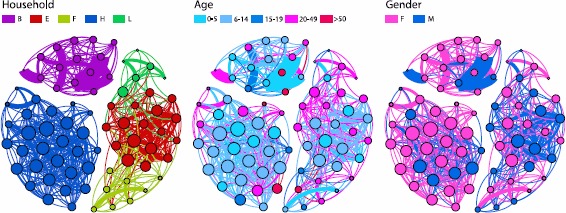
**Network of contacts between household members aggregated over the whole study duration.** Nodes are color coded according to their household (left panel), their age (central panel), and gender (right panel). Nodes’ size is proportional to their degree. Edge thickness is proportional to the total time spent in contact by two connected individuals.

**Figure 5:**
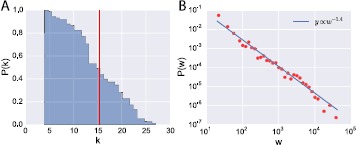
**Statistics of the aggregated contact network.** (**A**) Cumulative degree distribution $P(k)$ of the contact network aggregated over the whole study duration, *i.e.*, probability that a randomly chosen node has degree ≥*k*. The red line indicates the average degree value, $\langle k \rangle=15.3$. (**B**) Distribution of the weights of the aggregated contact network. The weight $w_{ij}$ of an edge *i*-*j* represents the total cumulative time spent in face-to-face proximity by individuals *i* and *j* during the whole study duration. The solid line is shown as a guide to the eye.

We also investigated the structure of the fully aggregated contact networks, considering both contacts within and between households. Figure 4 displays a pictorial representation of the contact network of the 5 households, where nodes are individuals and edges indicate the presence of at least one recorded contact during the study period. Nodes are color coded according to their household (panel A), age (panel B) and gender (panel C), and the size of each node *i* is proportional to its degree $k_{i}$. The edge thickness is proportional to the weight $w_{ij}$ that corresponds to the total amount of time spent in proximity by the two individuals. The contact network is formed by 75 nodes and 576 edges. The network is not fully connected but there are three connected components corresponding to households B and H, plus the aggregation of households E-F-L. The average degree of the network is $\langle k \rangle=15.3$, and the average clustering coefficient is equal to 0.79, indicating a high level of clustering (a random network with the same number of nodes and edges would have a clustering coefficient equal to 0.20). From panel B, it is possible to notice the high level of assortativity that characterizes the youngest age classes. The average degree of children aged 0-5 and 6-14 is 17.5 and 18.3, respectively, thus higher than the global average (see Table 2). It is also possible to observe the rather low variance of the degree distribution $P ( k )$ ($\mathit{CV}^{2} =0.13$), as shown in Figure 5 (panel A). The degree distribution extends between $k_{\mathrm{min}} =4$ and $k_{\mathrm{max}} =27$, and it is peaked around its average value. The observed low variance of the $P ( k )$ is in agreement with previous empirical studies on human contact networks [19, 24, 31]. On the other hand, the weight distribution is heterogeneous and decays approximately as a power law, with an exponential cut-off, as shown in Figure 5, panel B.

**Figure 6:**
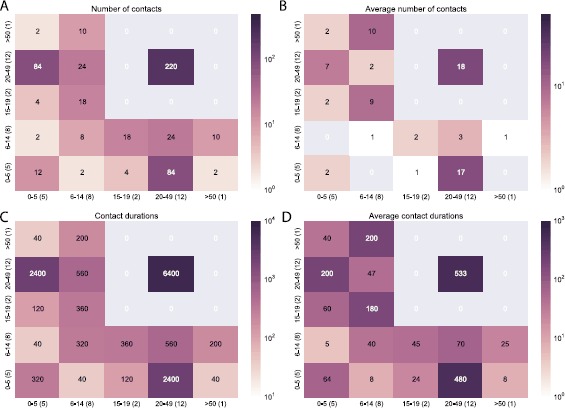
**Contact matrices giving the number (A)-(B) and cumulative duration (C)-(D) of contacts across households by age.** The left panels show the number of contacts (**A**) and corresponding durations (**C**) that individuals of age *i* (column index) had with individuals of age *j* (row index). The right panels show the average number (**B**) and duration (**D**) of contacts of an individual of age *i* with individuals of age *j*. Labels on the *x* and *y* axes report the age groups and the number of individuals in each group, in parenthesis. Durations are reported in seconds.

In the full contact network, there are 61 edges (11% of the total) connecting 28 members of different households (E, F and L). Only 3 (of 6) members of household L did not record contacts with members of other households (one Male 19 yo, one Female 12 yo, one Female 45 yo) but all members of household E and F had contacts with members of other households. As highlighted by the layout of Figure 4, panel A, members of household E had contacts with both members of households F and L, while there was only 1 contact between two individuals of the latter two households. It is important to notice that such inter-household contacts were recorded during short time windows for all the three households (see Figure S11 in Additional file 1). More specifically, all contacts between members of households E and L were recorded during two three hour intervals on two different days, and all contacts between members of households E and F were recorded between 11 am and 12 pm on one day, and between 9 am and 10 am in a following day. Interestingly, contact matrices extracted from inter-household contacts display some significant differences from those extracted from intra-household contacts, as shown in Figure 6. Although children aged less than 16 are present in the inter-household contact matrix there is no significant assortativity among them, and most of the contacts and the total time spent in proximity are recorded between adults (aged 20-49).

### Longitudinal analysis and stability of daily contact patterns

**Figure 7:**
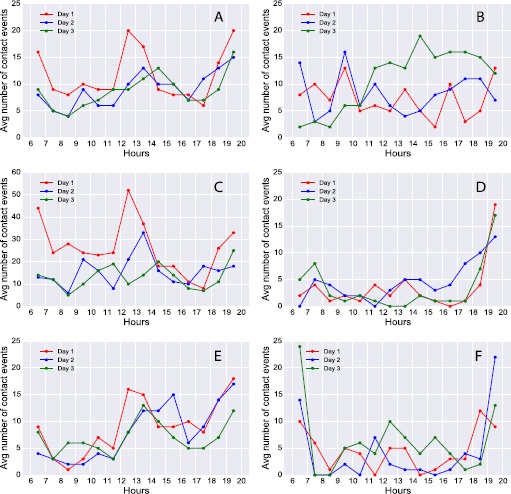
**Timeline of contact activities.** Average number of contacts for each individual recorded every hour from 6 am to 8 pm, by day of experiment. Activity is aggregated over all households (panel **A**) and displayed by each household: household B (panel **B**), household E (panel **C**), household F (panel **D**), household H (panel **E**), household L (panel **F**). Only contacts within households are considered.

Figure 7 shows the temporal evolution of hourly average number of contacts per individual, over three days of the study from 6 am to 8 pm. Overall (A), contacts evolve over time in a similar manner on each of the three days. Contacts are numerous in the morning and decline up to noon, peaking again between 1 pm and 2 pm. Their numbers experience a decline again in the afternoon up to about 5 pm and then a sharp increase during the night up to 8 pm. Panels B, C, D, E, F represent households B, E, F, H and L respectively. All households except B display a regular contact pattern similar to the description above over the three days.

**Figure 8:**
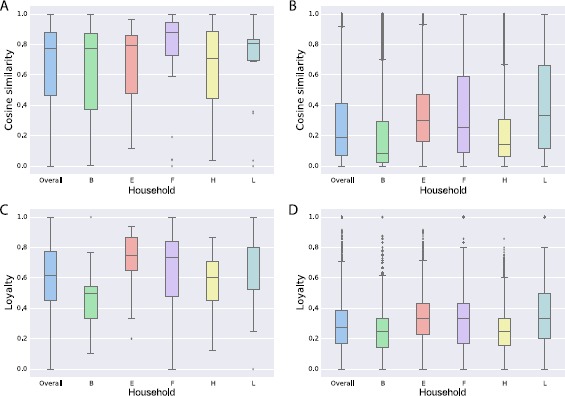
**Cosine similarity and loyalty of ego networks.** Distributions of cosine similarities (**A**) and loyalties (**C**) measured for each node of the full contact network (overall) and of each household. Distributions are obtained by measuring the cosine similarity and the loyalty of each node’s neighborhood, for each pair of days. Distributions are compared to those obtained from a randomized version of the contact networks (1,000 realizations for each null model). We consider two types of null models: weight reshuffling (**B**) and rewiring (**D**). Box plots indicate the median and the IQR of the distributions. The whiskers correspond to the $1.5 \times \mathrm{IQR}$ of the distributions.

We then looked at the daily contact networks, obtained by aggregating all the contact events measured between 6 am and 8 pm of each day, for each household. To assess the changes in the contacts of each household member, we computed the loyalties and the cosine similarities between the neighborhoods of each node in each pair of daily networks. The distributions of similarities and loyalties are shown in Figure 8, aggregated for all households and by each household. Median values of the distributions are quite close to 1 and the IQRs all lie above 0.4, with the exception of the distribution of household B. More specifically, the median values of the cosine similarity all vary between 0.7 and 0.9 (panel A), and the median values of the loyalty vary between 0.5 and 0.8 (panel C), indicating a substantial stability of individual contact patterns across days.

To better understand how much these values can be considered ‘large’, given the relatively small size of the networks under study, we compared the values of cosine similarity and loyalty to the ones measured on a set of different null models, *i.e.*, randomized versions of the contact networks. More in detail, we considered two types of null models: one in which the topology of the network is unchanged but the weights of the network are reshuffled among the edges (Figure 8, panel B) and one in which the network edges are placed at random between the nodes of the network (Figure 8, panel D). We computed the cosine similarity distributions on 1,000 realizations of the first null model and found much smaller median values, varying between 0.1 and 0.3, at the global level and for each household, than observed in the original network (IQRs ranging between 0.1 and 0.6). Similarly, we computed the loyalty distributions on 1,000 realizations of the second null model and found smaller median values, varying between 0.2 and 0.4, for all the contact networks under study.

## Discussion

The use of wireless proximity sensors (‘tags’) to collect data on close proximity interactions relevant for infectious disease transmission has gained significant ground. Whereas paper diary studies define a contact as a direct physical touch or conversation between co-located individuals, a contact event is a continuous set of 20-second interactions between two tags without a 20 second break. Tags capture the dynamics of contacts by collecting high resolution temporal data without influences of recall bias in paper diaries, and are relatively easy to deploy in various settings including hard to reach populations such as children and the elderly in rural areas [21]. Data collection using tags has mainly been done in closed settings of developed countries such as schools [30, 31, 35, 48], hospitals [35, 40], conferences [37, 49, 50], offices [32], universities [29] and a museum [51]. However, no work has to date been undertaken in household settings. Moreover, the demographic, social and mobility structures of individuals in developing countries may differ significantly compared to these settings, and there is no recorded use of wearable proximity sensors in developing country contexts. The primary aim of this study was to estimate social contact patterns and networks in a low resource developing country setting. This also provided an opportunity to assess social factors and logistical challenges that influence the deployment of wearable sensors for contact detection in a rural developing country community. We report here the first study to use close proximity sensors within the household setting, undertaken in a rural low-income community in coastal Kenya.

In the design of social network studies to inform mathematical models of infectious disease spread, it is important to note the contextual differences between developed and resource poor settings. Previous studies in institutions (schools [33, 34, 38], hospitals [30, 36, 52], conferences [37], museums [49]) on use of wearable sensors to detect close proximity interactions have mainly focused on challenges in computation of statistical measures of networks. Little attention has been paid to social and logistical challenges in conducting such studies even more so in environments such as school and households with high mixing rates between participants and non-participants. Intensive community engagement that involved local administrators, opinion leaders and entire households facilitated community entry and ensured that key messages were restructured and easily understood. Focus group discussions at the onset of this study provided invaluable input on study procedures such as how to ensure the devices were acceptable to participants and minimize curiosity in other community members. Most residents, particularly women, normally hang mobile phones on lanyards around their necks. We took advantage of this and requested participants to carry the devices in pouches around their necks to minimize attracting attention to themselves. Bias due to non-compliance, such as not wearing a tag or picking a different tag in the morning, and behavior change were potential problems. Although an exit interview was not conducted to assess the effects of the recommendations from the focus group discussions, there was relevant anecdotal evidence that these biases did not affect the data collection significantly as suggested in a similar study in a primary school [53]. This could be attributed to familiarity as individuals become accustomed to wearing the device over time. To avoid other unobserved effects such as exchange of tags, future studies in high-density populations, where participants keep the tags over several days, could resort to other simple measures to identify unique tags for each participant such as using different colour pouches for each participant.

We collected data continuously over a full 24 hours each day unlike other studies that did not collect data outside normal school or work hours [32, 34]. However, all nighttime (8 pm-6 am) contacts were disregarded during analysis due to inconsistent spikes in data that suggested heightened interaction between participants (probably the result of removal and storage of devices together). In general, family members congregate in the morning, over lunch hour and again in the evening. This is suggestive of normal human social behavior in rural Kilifi, whereby members of a household congregate for breakfast, lunch and when everyone returns home from school, work or other engagements. Combining this with time-use data would have provided further insight into the fluctuations in contacts during the day, particularly by elucidating where individuals spend their time and suggesting other potential contacts they made with people outside the study. While it may be possible to issue tags to all participants at the same time in closed settings such as school or health institutions, studies involving several households pose several challenges. For example, it was not feasible to collect household data contemporaneously across all five households due to three main reasons: there were fewer tags compared to the total number of household members, the 5 households were not selected for their close proximity and it took approximately half a day per household to issue tags to all residents, and lastly the need to recharge devices after each use at the research office due to lack of electricity in the selected households.

Overall, at a coarse-grained level, the observed structure of the household contact matrices is consistent with the assumption of a strong age assortativity, as measured from self-reported contact diaries [16] and often integrated into mathematical epidemic models [54]. Many contacts are observed among children, and between children aged <5 years and adults, while teenagers and elderly individuals tend to have lower contact frequencies. On the other hand, contact matrices are not fully captured by a simple random mixing assumption, that is typical of large synthetic contact networks built from socio-demographic data [8]. More sophisticated modeling approaches, such as latent variables models as proposed in [55], could be more suitable to fit the observed contact patterns within households.

Results further indicate a strong variability of contact durations in households, characterised by heavy-tailed distributions, and confirm the presence of ‘universal’ characteristics of contact patterns also in this setting, as previously measured in schools [34, 38] and hospitals [35]. We did not find any significant difference by gender in the durations of contacts, with the distributions being essentially equal to the aggregated one. It is worth noting, however, that we did not investigate the presence of gender homophily, which has been observed in primary schools [44], where the likelihood of infection transmission in school children of the same gender is high due to gender assortativity of contacts [41]. Distributions of contact durations did not vary significantly from day to day, suggesting the presence of robust and repetitive contact patterns on a daily temporal scale. It is important to note that this study was conducted over three weekdays only, and thus we might expect to observe differences in network structure in a longer study that would incorporate weekends. In line with the observations of previous SocioPatterns measurements [34, 36, 38, 56], the probability distribution of contacts (Figure 2) is suggestive of many brief contacts and few long-lasting contacts, whose probability is however not negligible. For communicable diseases whose transmissibility depends on duration of contact, this may play a key role in defining the probability of transmission given a short or long contact duration [57]. Recent analysis of time use and contact data suggests there is a minimum ‘suitable duration’ of exposure for transmission to occur, dependent on the transmissibility of the infection [58]. However, further investigation is required to understand the characteristics of contact events associated with transmission of common childhood respiratory infections such as viral pneumonia by combining a variety of methods, including longitudinal surveillance of contacts and microbiological data.

As reported in previous studies [32, 59], contact patterns may exhibit important heterogeneities at different time scales relevant for disease transmission. At the finest scale of minutes and hours, our data display strong fluctuations driven by the circadian activities of individuals, as one may easily expect. Whether such fluctuations should be expected also at the daily or longer time scale, remains an open question. To address this issue, we performed a longitudinal analysis of the contact networks extracted from the tags, with the main goal of measuring potential similarities between contacts measured from households on different days, keeping in mind the constraints imposed by the short study duration. These results indicate that the observed contact patterns of each household member were significantly similar from day to day; at the level both of contacted individuals (as measured by the loyalty) and of the durations of time spent in contact with different individuals (as measured by the cosine similarity). Overall, within-household contacts appear to be highly stable and repetitive across single days, thus suggesting that a short data collection period of a few days could be sufficient for an accurate description. Such information is relevant to understand how much a single experimental day can be considered representative of the typical contact patterns within a household and how much data gathering would be needed to obtain a comprehensive picture of the full contact network. On the other hand, contacts across households, mainly driven by adults who could thus act as bridges in transmission of communicable diseases from one household to the other, appear to be irregular and quite difficult to capture during a time window of a week or less. While between-household contacts could be a significant driver of infectious diseases, the sample size does not allow obtaining any definitive insights on the mixing behaviour of the population in general. Furthermore, our results support the role of children in transmitting respiratory infections within the household [45, 60, 61].

It is important to highlight some key limitations of the present study. It was not possible to collect data from all 5 households contemporaneously due to the limitations mentioned. Indeed, it is impossible to saturate an entire community with the tags, thus limiting the ability to reconstruct full networks. However, for future household studies, recruitment could be conducted in household clusters and tags issued on the same day for all participants to ensure contemporaneous data collection. Currently, a smaller and lighter wireless sensor that should enable younger children to participate is available. The new sensor also has a longer battery life and bigger storage space, thus enabling data collection over longer periods, which is especially useful in hard to reach populations where there is no electricity connection in households (www.get.openbeacon.org). Data from a quarter of the tags were disregarded due to both tag malfunction and human influences such as storing tags together, *e.g.*, during nighttime. While it may not be possible to detect tag malfunction during data collection, future studies can minimize human error by proper training on how to use and store the tags especially when collecting data for longer periods. There were few individuals aged 16-19 years (8%) at the households resulting in few contacts being recorded with this age group. Residents of this age were away at boarding school or work, which can be alleviated by longitudinal studies incorporating both term and holiday times as well as weekends. This represents a highly mobile age group whose interactions may be important in transmission of respiratory infections and hence a potential target in intervention design. However, debate still remains on how large and long a study should be to capture an accurate representation of the dynamism of social networks and contacts [62, 63]. Furthermore, the study population was composed of students, subsistence farmers, small business operators and fishermen, which is typical of the coastal setting in Kenya. As such, results cannot be generalized to urban locations or other inland locations that have different social, economic, cultural and demographic characteristics. Future studies should consider higher coverage for example, in rural and urban household clusters rather than entire communities, to generate more generalizable insights into network characteristics of different regions. Lastly, collecting temporal contextual data such as time use and location would potentially explain the structure of daily contacts and why day-to-day differences exist in some cases or between households.

Our approach used electronic proximity sensors to collect social contact data from households in a rural setting. The results suggest important differences in within and between household contacts, with individuals of different ages driving the frequency and distribution of contacts in this setting. This should be a focus of future investigation. Despite involving only 5 households, insights from this pilot study can be valuable to the design of larger community-wide studies, such as how long to conduct data collection or how many people to involve. Continued work of this nature is important in understanding the temporal dynamics of household contacts and networks with a view to informing the design of intervention strategies.

## Electronic Supplementary Material

Below is the link to the electronic supplementary material.

**Supplementary information.** Contact matrices for each household. Contact matrices from a synthetic model. Inter-household contact timeline. (pdf)
