# Supplementary material for: Quantifying social contacts in a household setting of rural Kenya using wearable proximity sensors
Source: EPJ Data Sci. 2016 Jun 14;5:21. doi: 10.1140/epjds/s13688-016-0084-2 (PMC4944592; doi:10.1140/epjds/s13688-016-0084-2)
Supplement: Supplementary file 1 — Supplementary information. Contact matrices for each household. Contact matrices from a synthetic model. Inter-household contact timeline. (pdf) [file 13688_2016_84_MOESM1_ESM.pdf]

Quantifying social contacts in a household setting of rural Kenya using wearable proximity sensors
Supplementary Information

M. C. Kiti M. Tizzoni T. M. Kinyanjui D. C. Koech
P. K. Munywoki M. Meriac L. Cappa A. Panisson A. Barrat
C. Cattuto D. J. Nokes

1 Contact matrices for each household

Here, we present the contact matrices computed with the proximity sensor tracks for each household. In Figures S1 - S5, the left panel shows the total number of contacts that individuals of age i (column-index) had with individuals of age j (row-index) over 3 days. The right panel shows the cumulative duration of contacts between individuals of age i and individuals of age j. Labels on the x and y axes report the age groups and the number of individuals in each group, in parenthesis. Durations are reported in seconds.

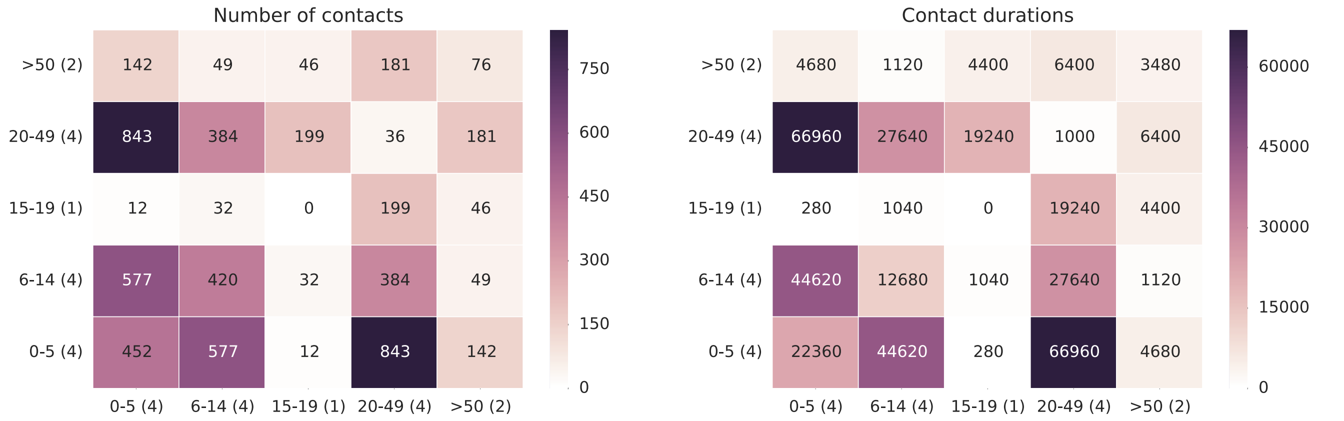

Figure S1: **Contact matrices giving the number and cumulative duration of contacts in household B, by age.**

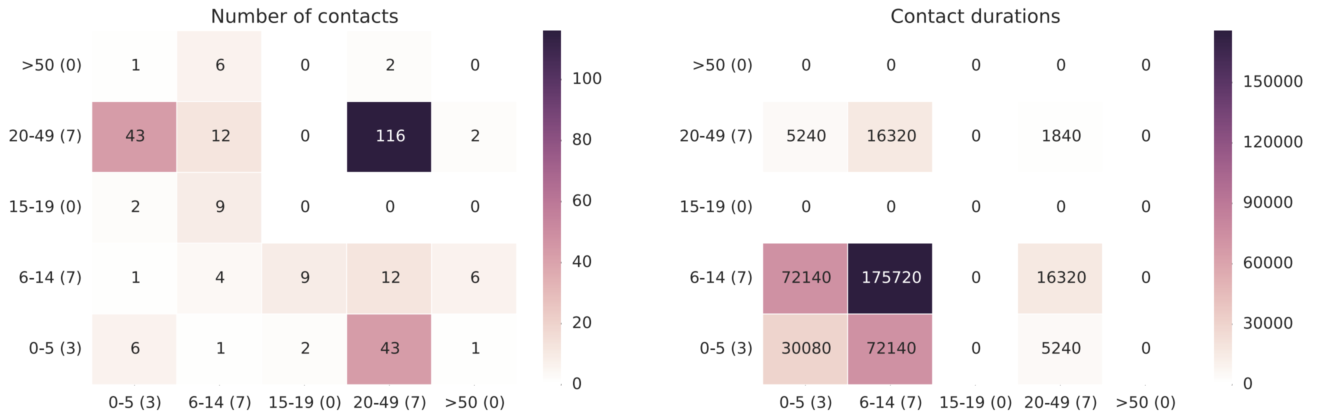

Figure S2: **Contact matrices giving the number and cumulative duration of contacts in household E, by age.**

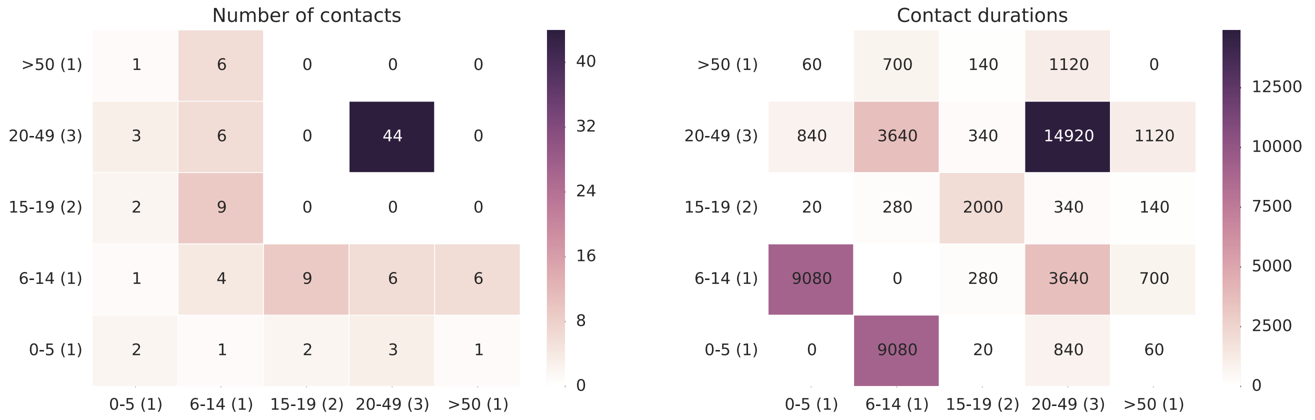

Figure S3: **Contact matrices giving the number and cumulative duration of contacts in household F, by age.**

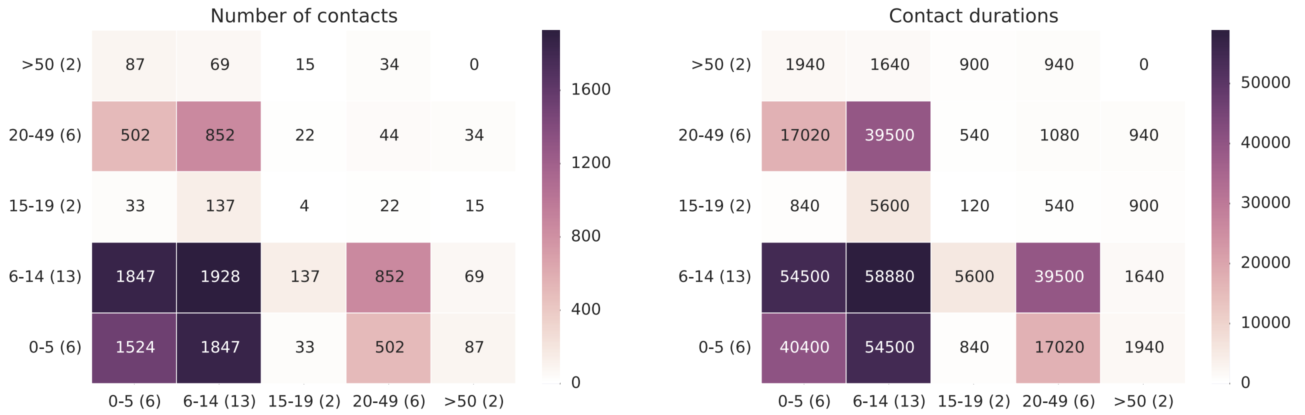

Figure S4: **Contact matrices giving the number and cumulative duration of contacts in household H, by age.**

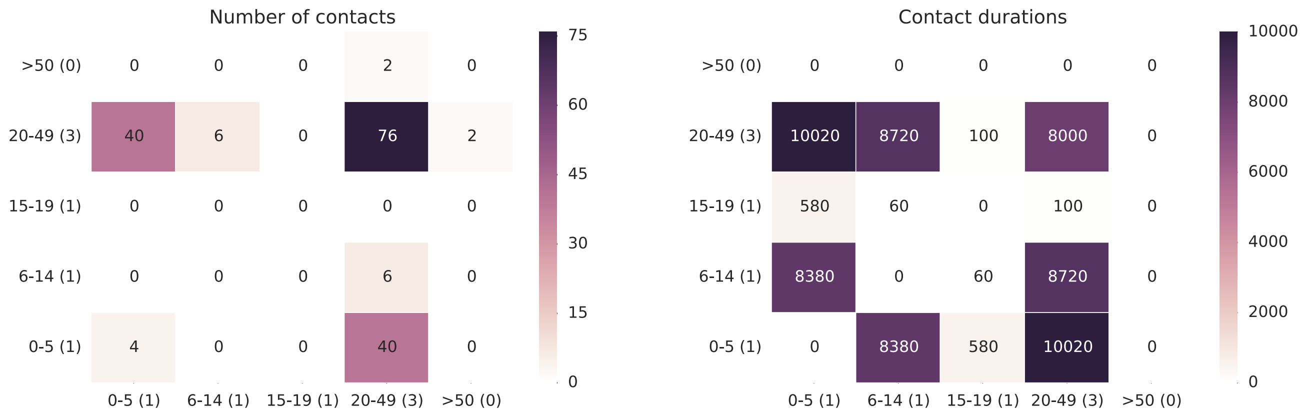

Figure S5: **Contact matrices giving the number and cumulative duration of contacts in household L, by age.**

2 Contact matrices from a synthetic model

Here, we compare the contact matrices computed with the proximity sensor tracks and the contact matrices that would be obtained assuming a fully random mixing in each household. More specifically, we compute such synthetic matrices using the approach of Fumanelli *et al.* [1].

More specifically, we compute the frequency of contacts within any household H, between individuals of ages i and j, denoted as f_{ij}^H using the formula:

$$f_{ij}^H = \begin{cases} \frac{1}{n_i^H} \sum_{1 \leq k \leq n_i^H} \frac{h_j^{(k)} - \delta_{ij}}{\nu_H^{(k)} - 1} & \text{if } n_i^H > 0 \\ 0 & \text{otherwise,} \end{cases} \tag{1}$$

where n_i^H is the number of individuals of age i in household H, $\nu_H^{(k)}$ is the size of the household, $h_j^{(k)}$ is the set of individuals of age j, living in H.

In Figures S6 - S10, the left panel shows the fraction of the total number of contacts that individuals of age i (column index) had with individuals of age j (row index) over 3 days, as measured by proximity sensors. The right panel column shows the expected frequency of contacts, f_{ij}^H between individuals of age i and individuals of age j based on Equation 1. Labels on the x and y axes report the age groups and the number of individuals in each class, in parenthesis.

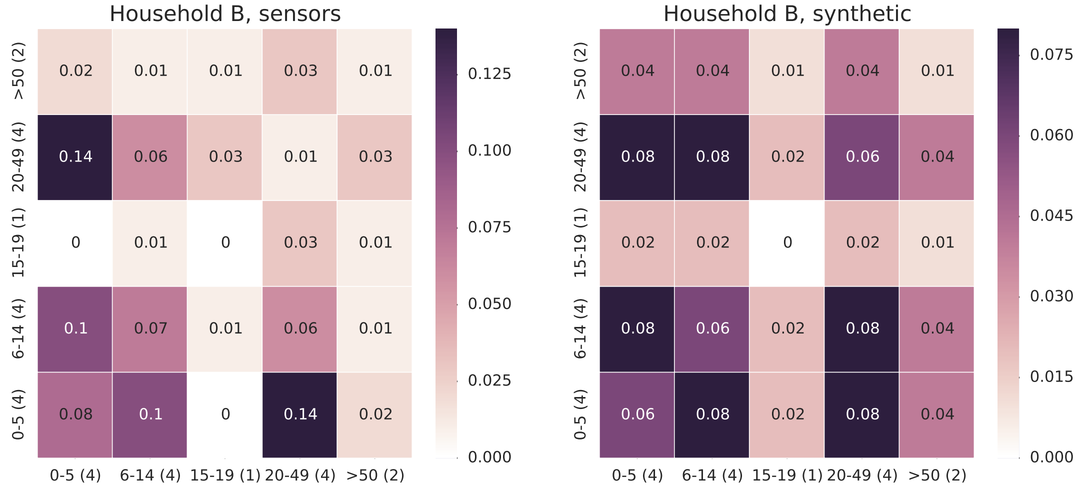

Figure S6: **Comparison between contact matrices measured by proximity sensors and synthetic contact matrices for household B.**

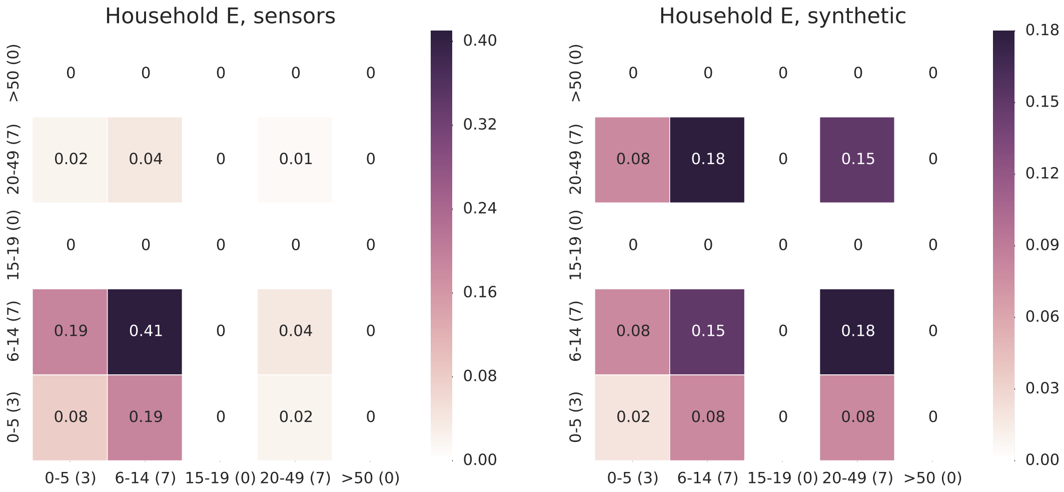

Figure S7: **Comparison between contact matrices measured by proximity sensors and synthetic contact matrices for household E.**

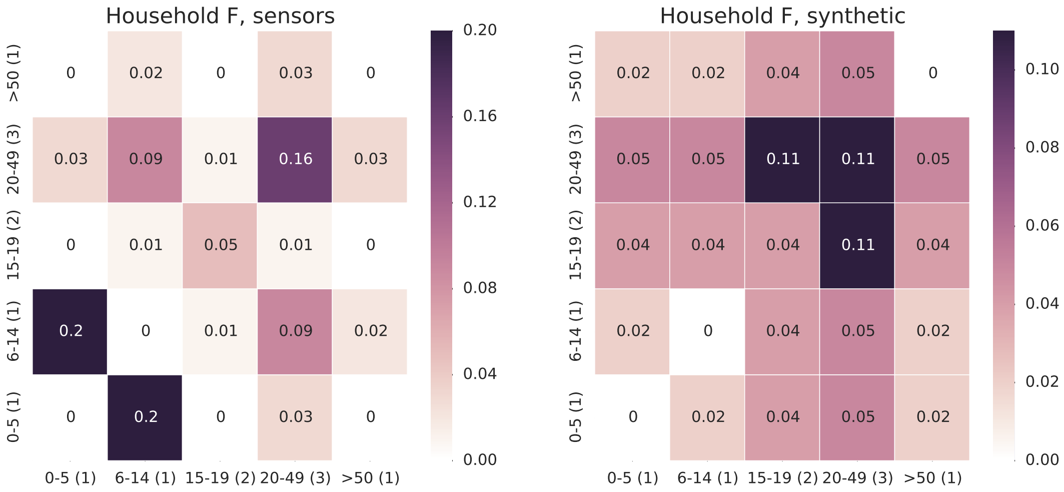

Figure S8: **Comparison between contact matrices measured by proximity sensors and synthetic contact matrices for household F.**

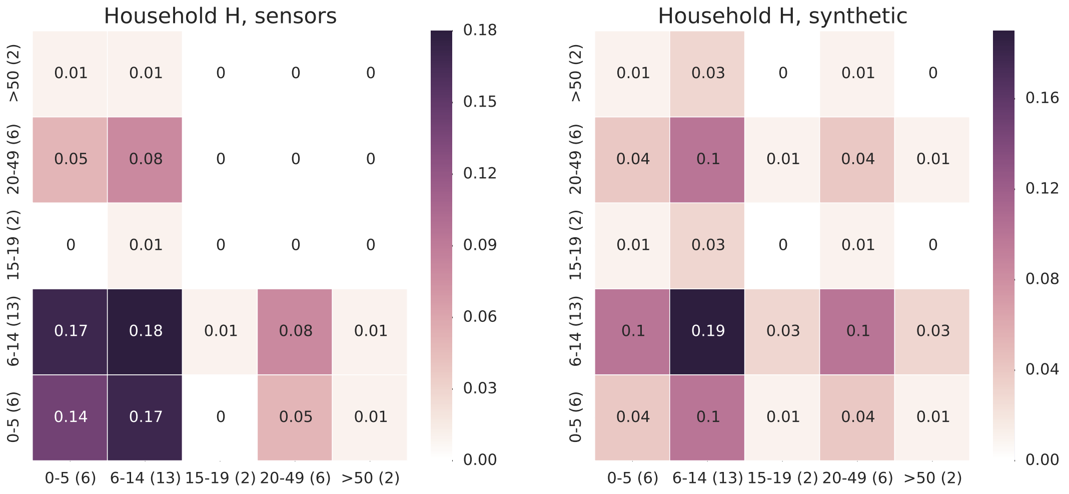

Figure S9: **Comparison between contact matrices measured by proximity sensors and synthetic contact matrices for household H.**

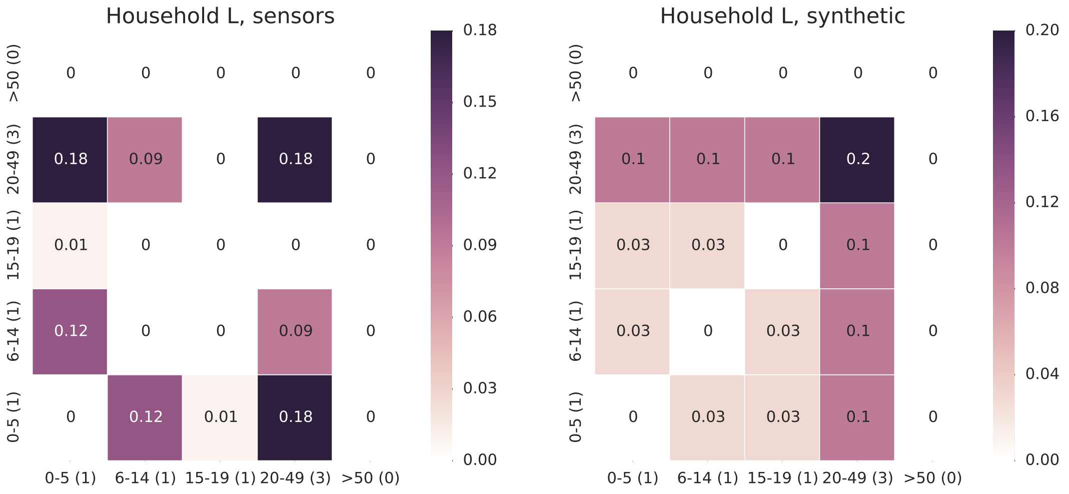

Figure S10: **Comparison between contact matrices measured by proximity sensors and synthetic contact matrices for household L.**

3 Inter-household contact timelines

Here, we show the time of the day when contacts between members of different households have been recorded. Inter-household contacts have been recorded only in 4 different days. Figure S11 displays the total number of contacts recorded every hour between members of households E and L (panel A) and households E and F (panel B).

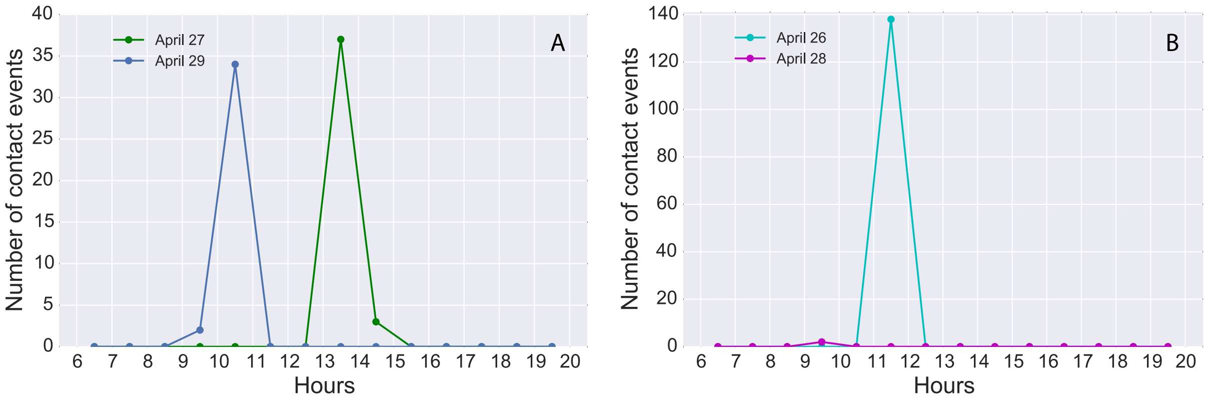

Figure S11: **Timeline of contact activities across households.** Total number of contacts recorded every hour from 6am to 8pm, by day of experiment, between members of different households. Panel A shows contacts between members of household E and L, panel B shows contacts between members of household E and F.

References

[1] L. Fumanelli, M. Ajelli, P. Manfredi, A. Vespignani, and S. Merler. Inferring the structure of social contacts from demographic data in the analysis of infectious diseases spread. *PLoS Comput Biol*, 8(9):e1002673, 2012.
